# Trauma simulation training: a randomized controlled trial ­evaluating the effectiveness of the Imperial Femoral Intramedullary Nailing Cognitive Task Analysis (IFINCTA) tool

**DOI:** 10.1080/17453674.2018.1517442

**Published:** 2018-10-17

**Authors:** Rahul Bhattacharyya, Kapil Sugand, Bilal Al-Obaidi, Ian Sinha, Rajarshi Bhattacharya, Chinmay M Gupte

**Affiliations:** 1 MSK Lab, Imperial College London, Charing Cross Hospital Campus, Laboratory Block, London;; 2 St Mary’s Hospital, Imperial College Hospitals NHS Trust, London, UK

## Abstract

Background and purpose — Cognitive task analysis (CTA) has been used extensively to train pilots and in other surgical specialties. However, the use of CTA within orthopedics is in its infancy. We evaluated the effectiveness of a novel CTA tool to improve understanding of the procedural steps in antegrade femoral intramedullary nailing.

Material and methods — *Design:* A modified Delphi technique was used to generate a CTA from 3 expert orthopedic trauma surgeons for antegrade femoral intramedullary nailing. The written and audiovisual information was combined to describe the technical steps, decision points, and errors for each phase of this procedure *Validation:* A randomized double-blind controlled trial was undertaken with 22 medical students (novices) randomized into 2 equal groups. The intervention group were given the CTA tool and the control group were given a standard operative technique manual. They were assessed using the validated “Touch Surgery™” application assessment tool on femoral intramedullary nailing.

Results — The pre-test scores between the two groups were similar. However, the post-test scores were statistically significantly better in the intervention group compared with the control group. The improvement (post-test median scores) in the intervention group compared with the control group was 20% for patient positioning and preparation, 21% for femoral preparation, 10% for proximal locking, and 19% for distal locking respectively (p < 0.001 for all comparisons).

Interpretation — This is the first multimedia CTA tool in femoral intramedullary nailing that is easily accessible, user-friendly, and has demonstrated significant benefits in training novices over the traditional use of operative technique manuals.

There are significant strains on surgical training due to working regulations, increased malpractice cases, and reduced training time in the operating theatre (Philibert et al. 2002, RAD 1382 Consensus Statement 2008). Innovative adjuncts to the traditional apprenticeship model are required to reduce the learning curve and improve utilization of theatre training time.

Previous research has highlighted the benefits of virtual reality (VR) and cadaveric simulation in orthopedic training (Cannon et al. 2014, Rebolledo et al. 2015, Sugand et al. 2015a, Camp et al. 2016, Tomlinson et al. 2016). However, these simulators are expensive and are not readily accessible to trainees worldwide. Karam et al. (2013) showed that 25% of training programs in the USA do not have a dedicated simulation facility and 87% of training program directors reported a lack of sufficient funds as the main barrier.

The burden of orthopedic trauma is greater than ever before with major trauma now affecting the elderly population in addition to the high-energy injuries in the younger age group (Kehoe et al. 2015). In this setting, newer, innovative, and easily accessible training adjuncts to the traditional apprenticeship model are necessary to help train competent orthopaedic trauma surgeons.

It is well established that cognitive learning of motor skills is key to undertaking a successful practical procedure (Spencer 1978, Flin et al. 2007, Wingfield et al. 2015, Wallace et al. 2017). Wingfield et al. (2015) defined cognitive task analysis (CTA) as a process by which experts provide complex information to trainees in a logical and simplified manner. This makes it easier to visualize and digest procedural steps. It has been extensively used to train pilots (Wingfield et al. 2015), and in other surgical specialties (Sullivan et al. 2008, Luker et al. 2008, Arora et al. 2011, Smink et al. 2012). However, it is relatively new to orthopedic training. Bhattacharyya et al. (2017) reported the effectiveness of the first CTA tool in orthopedic training on diagnostic knee arthroscopy. There are no studies in the literature that have investigated the efficacy of CTA to teach orthopedic trauma procedures, which forms a major part of the orthopedic training curriculum, especially in the early years of training (BOTA Collaborators and Rashid 2018).

We evaluated the effectiveness of a novel CTA tool to improve understanding of the procedural steps in antegrade femoral intramedullary nailing.

## Null hypothesis

There was no difference in objective test scores between novices learning the procedural steps of antegrade femoral intramedullary nailing from our CTA compared with the op-tech manual.

## Material and methods

We first designed a CTA tool and then evaluated its effectiveness to teach the procedural steps.

Design of the Imperial Femoral Intramedullary Nailing Cognitive Task Analysis (IFINCTA) tool

## Selection of experts

The inclusion criteria for experts were: consultant, fellowship-trained trauma and orthopedic surgeons, who practice trauma as part of their routine practice in a designated major trauma centre in the UK. Experts who matched the above criteria and were prepared to devote the time required for the Delphi process were selected to design the CTA. They were all male; median age 43 years (41–45); median years in consultant practice: 8 (4–8); median years in formal teaching: 8 (4–8). 2/3 experts were part of the group who received the grant from the AO Foundation for developing simulation techniques in trauma. However, the content of this CTA tool had no influence on the results of the validation part of the study as the experts were not involved in objectively scoring the participants in the trial.

## Procedural steps and Delphi technique

The modified Delphi technique was used to generate the final written information of the IFINCTA tool. This technique involved a combination of independent analysis and “face to face” review from the 3 experts over 3 rounds.

Round 1: The experts were interviewed independently to generate a list of technical steps, cognitive decision points, and potential errors and solutions that are applicable to an antegrade femoral intramedullary nailing procedure. All the technical steps and decision points that were common to the 3 experts were identified in round 1.

Round 2: The steps that were common to 2 experts or exclusively generated from 1 expert were listed separately. This list was reviewed by the experts independently and they either agreed or disagreed with these steps. The steps agreed upon by all experts in rounds 1 and 2 were listed at the end of round 2.

Round 3: This involved a “face to face” meeting of the expert panel where they reviewed all the steps that were agreed upon in rounds 1 and 2. Only those steps that had 100% consensus from all 3 experts at the end of the 3 rounds were included in the final CTA.

## Audiovisual component

1 of the experts in our study recorded a video of an antegrade femoral intramedullary nailing procedure for a diaphyseal mid-shaft femoral fracture. This video was divided into separate clips reflecting each of the 7 phases. A voiceover was recorded later using a “free recall” technique with the same expert. In this technique, the same expert recorded an audio clip describing each phase of the procedure by watching the video clips retrospectively. This was superimposed on the video clips. The other 2 experts reviewed this video and a consensus was reached regarding the final content of the video so that it accurately reflected the written CTA master template.

## Creation of the IFINCTA tool

**Figure 1.**
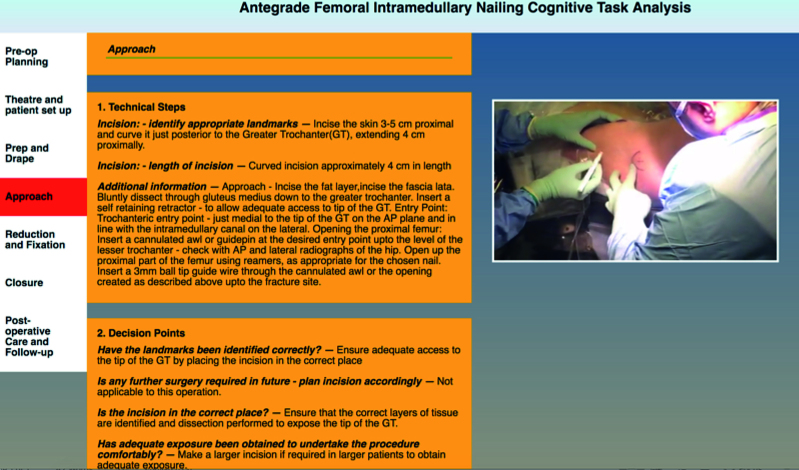
Snapshot of the IFINCTA tool showing the written information of the CTA on the left and the multimedia video clips and audio recordings on the right for the “approach” phase of the procedure.

The written information (Delphi method), video clips, and audio recordings were input into a “wizard” tool developed in our unit. This is a web-based tool used by experts to create CTAs. This led to the design of the IFINCTA tool (Figure 1). This tool has a split-screen format. The left side has all the written information on the technical steps, cognitive decision points, and potential errors and solutions. The right side includes the multimedia modalities (video clips, audio voice recordings).

### Experimental design to evaluate the effectiveness of the IFINCTA tool

This was a double-blinded, randomized controlled trial.

### Participants

*Inclusion criteria:* Undergraduate medical students having observed 5 or fewer antegrade femoral intramedullary nailing procedures, neither having performed this procedure nor being familiarized with similar CTA tools beforehand.

*Exclusion criteria:* Graduates, having performed this procedure, having been exposed to “Touch Surgery^TM^” in the past, and observing more than 5 antegrade femoral intramedullary nailing procedures.

*Recruitment:* 30 undergraduate novices initially registered their interest in participating and completed a questionnaire assessing their experience in femoral intramedullary nailing. 8 novices could not attend due to other commitments coinciding with the timing of the study. The remaining 22 novices all matched the inclusion criteria and completed the study. All participants gave written consent to participate in the study. They were recruited between October 2016 and April 2017.

### Randomization

The participants were randomized into 2 equal groups (n = 11) using the random number generator function on Microsoft Excel (Microsoft Corp, Redmond, WA, USA). This generated random numbers from 1 to 22. These numbers were stored in concealed envelopes prior to being assigned to either the intervention or the control group by a person in the lab who was not directly involved in conducting the trial.

### Trial

“Touch surgery™” application assessment tool

We used the “Touch Surgery™” application (Kinosis, London, UK) assessment tool for femoral intramedullary nailing validated by Sugand et al. (2015b). This is a cognitive, interactive VR application that utilizes computer animation to explain the operative steps of a surgical procedure. It is available on mobile and smart devices (free of cost). It has separate learning and assessment tools. In our study, we only used the validated assessment tool for 4 modules of this procedure (1) patient positioning and preparation, (2) femoral canal preparation, (3) proximal locking, (4) distal locking and closure). Points were scored for single best answer questions for each step of the procedure and for virtually completing manual steps (incision, drilling etc.) using finger-swipe interactions on the screen (Figure 2). The final score for each module is automatically calculated by the software and is scored as a percentage with no negative marking (Sugand et al. 2015b).

Figure 2.“Touch Surgery™” assessment tool for antegrade femoral intramedullary nailing: (left panel) demonstrating single best answer questions for cognitive decision making; (right panel) showing the digital swipe interactions required to complete the procedure.

**Figure:**
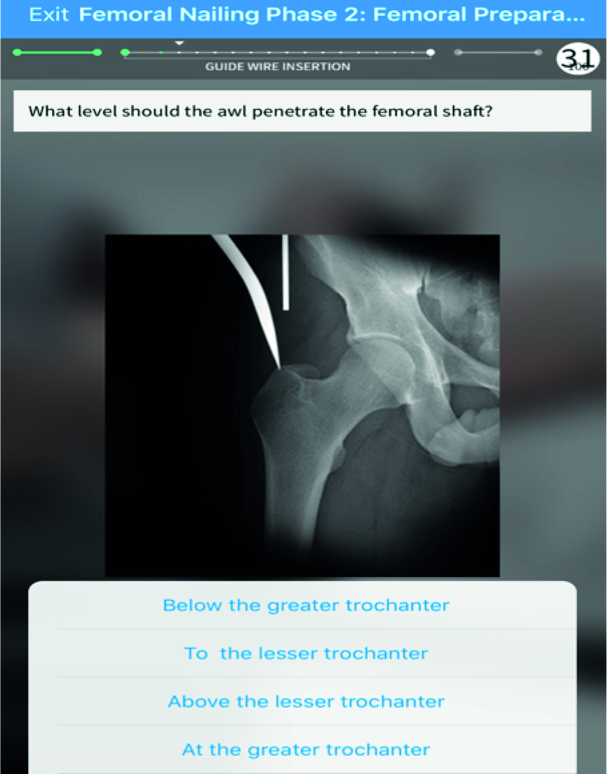

**Figure:**
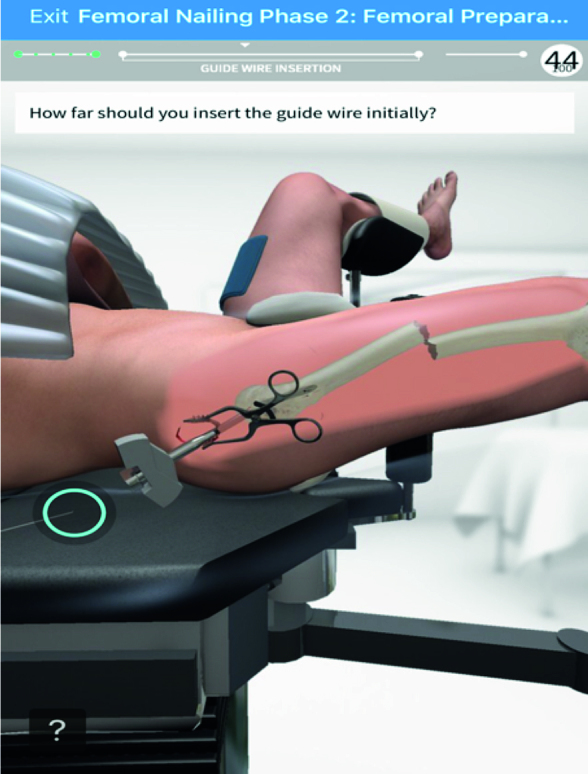

**Figure 3.**
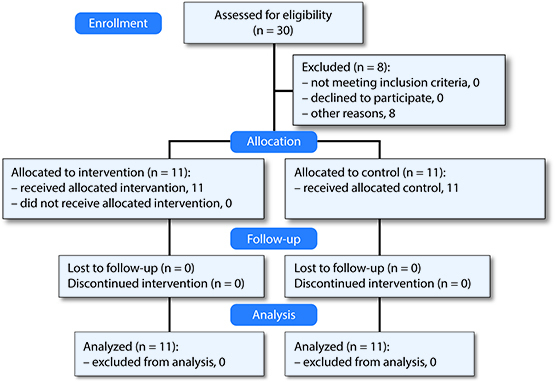
CONSORT diagram showing recruitment and follow-up of participants.

## Data collection

*Intervention and control groups:* The intervention group were given the IFINCTA tool developed in this study and the control group were given a standard operative technique manual for this procedure, which is representative of a common current method used by learners prior to performing this procedure in theatre.

*Objective testing:* The participants were supervised during the assessments by an independent research supervisor and were blinded to whether they belonged to the intervention or the control group. All participants completed a pre-test for analysis of baseline scores to ensure that the groups were homogeneous at baseline. They were then provided with their respective learning tools for 1 week and instructed to use them for as long as necessary in order to feel prepared before returning for the post-test. We analyzed the mean time (minutes) for which the participants in each group used their learning tools. At the end of the week the students completed the post-test using the same assessment tool. All testing occurred in the college laboratory facilities.

Primary outcome measures:

- The difference in the post-test scores between the intervention and the control groups.
- The difference between the pre-test and the post-test scores in the intervention and the control groups.

*Subjective testing (secondary outcome measure):* The students in the intervention group (n = 11) rated the IFINCTA tool on 5 parameters (Table 1) using the Likert rating scale (1 = strongly disagree, 2 = disagree, 3 = neither disagree nor agree, 4 = agree, 5 = strongly agree). The 3 expert consultant orthopedic trauma surgeons who designed the IFINCTA tool agreed on these parameters.

**Table 1.** IFINCTA tool rating parameters and rating analysis

No.	Parameter	Agreed^a^
1	The tool was easy to us0065	10/11
2	The tool made the procedure easy to understand	11/11
3	The written, visual and audio modalities being simultaneously present was useful	11/11
4	It would be beneficial to use this tool prior to attending an operating theatre session on femoral intramedullary nailing	11/11
5	You enjoyed using this tool	9/11

a > 3 on Likert scale

### Statistics

*Sample size calculation:* An a priori power calculation was conducted using mean percentage scores from a previous pilot study whereby the intervention group scored 80% vs. the control group which scored 55% (SD 19) in module 2 of the assessment tool with alpha =5% and beta =20% resulting in power =80%. The sample size resulted in a minimum n = 9 per group. The difference of scores was also deemed significant according to the expert surgeons.

*Software:* The data obtained from the validation study were input into the SPSS software version 23. The analysis using histograms, QQ plots, skewness, and kurtosis data showed that the data were nonparametric.

*Results:* The median (interquartile range) scores for the 4 modules were calculated for both groups. The Mann–Whitney U test was used for independent data and the Wilcoxon signed rank test was used for paired data. A p-value of <0.05 was considered to be statistically significant.

### Ethics, registration, funding, and potential conflicts of interest

Ethical approval was obtained from our institutional Medical Education Ethics Committee (MEEC—reference number: 1617-08).

Funding was received from the AO Foundation, Switzerland, under the AO Strategy Fund Project “Multipurpose Virtual Surgical Simulator”. The AO Foundation currently collaborates with the Touch Surgery^TM^ group; however, there was no association between the design of the IFINCTA tool in this study and the Touch Surgery^TM^ femoral nailing module, which was developed earlier and independent of the AO Foundation. The authors declared no conflicts of interest.

## Results

### Participant demographics, baseline characteristics, and flow through the study (Figure 3)

All 22 medical students (median age 23 years, 17 males) who matched the inclusion criteria completed the study. The median age was 22.5 years. The median year of undergraduate studies was 4 (1–5) in the intervention group compared with 5 (1–5) in the control group. The median number of antegrade femoral intramedullary nailing procedures observed in the operating room prior to the study in the intervention group was 1 (0–5) compared with 1.5 (0–5) in the control group. The mean time (minutes) for which the participants used their learning tool in the intervention group was 55 (45–65) compared with 57 (35–70) minutes in the control group.

### ^‘Touch Surgery^™^’ objective assessment^

*Pre-test scores:* There was no statistically significant difference in the median pre-test scores between the intervention and the control groups to demonstrate homogeneity of participants, and avoiding selection bias (Table 2).

**Table 2.** Comparison of pre-test “Touch Surgery™” assessment scores between intervention and control groups. Values are median scores (interquartile range)

Module	Intervention (IFINCTA)	Control “op tech”	p-value^a^
1. Patient positioning and preparation	50 (40–55)	45 (25–55)	0.4
2. Femoral canal preparation	53 (50–59)	56 (38–62)	1
3. Proximal locking	57 (50–60)	50 (50–57)	0.4
4. Distal locking and closure	57 (53–63)	58 (51–63)	0.7

a Mann–Whitney U test for median difference in scores

**Figure 4.**
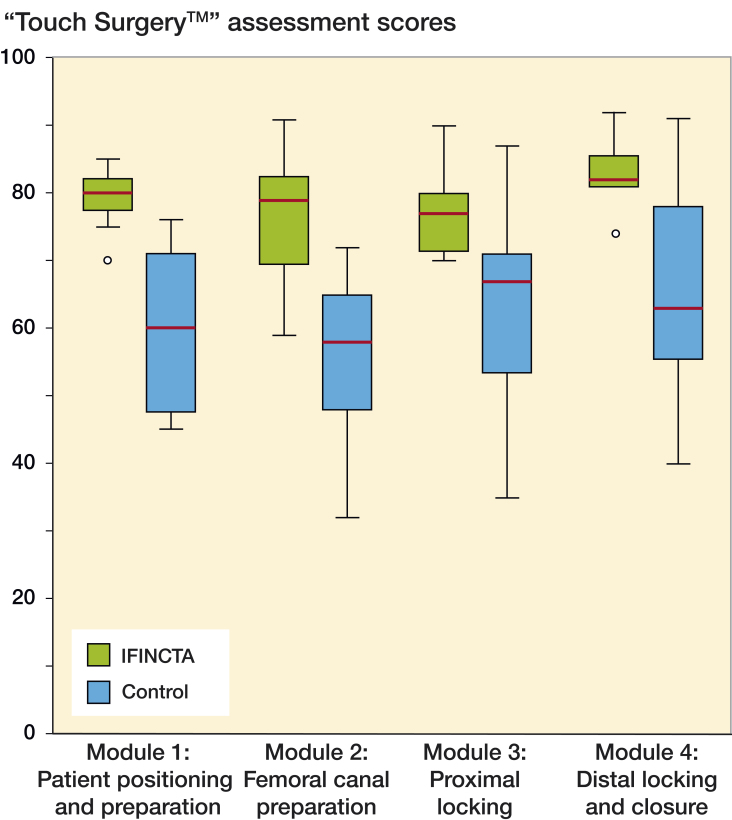
Box and whisker plot comparing post-test “Touch Surgery^TM^’ assessment scores between the IFINCTA and control groups for modules 1–4. Red lines are median values, boxes are interquartile ranges, whiskers are ranges of the data sets, and circles are 2 outliers.

*Post-test scores (primary outcome measure 1):* The post-test scores were statistically significantly better in the intervention group compared with the control group for all 4 modules in the “Touch Surgery™” assessment tool for antegrade femoral intramedullary nailing (Figure 4 and Table 3).

**Table 3.** Comparison of post-test “Touch Surgery™” assessment scores between intervention and control groups. Values are median scores (interquartile range)

Module	Intervention (IFINCTA)	Control “op tech”	Difference (%)	p-value ^a^
1. Patient positioning and preparation	80 (75–82)	60 (45–72)	20	< 0.001
2. Femoral canal preparation	79 (67–84)	58 (48–67)	21	0.001
3. Proximal locking	77 (70–82)	67 (52–72)	10	< 0.001
4. Distal locking and closure	82 (81–86)	63 (53–78)	19	< 0.001

^a^Mann–Whitney U test for median difference in scores

*Comparison between the pre- and post-test scores for both groups (primary outcome measure 2):* Both groups showed improvement in the post-test compared with the pre-test but this was much greater in the intervention group compared with the control group (Table 4).

**Table 4.** Comparison between pre-test and post-test scores for the intervention and the control groups. Values are difference between pre-test and post-test median scores

Module	Intervention (IFINCTA)	p-value^a^	Control “op tech”	p-value^a^
1. Patient positioning and preparation	30	< 0.001	15	< 0.001
2. Femoral canal preparation	26	< 0.001	2	0.4
3. Proximal locking	20	< 0.001	17	0.04
4. Distal locking and closure	25	< 0.001	5	0.04

a Wilcoxon signed rank test

### IFINCTA tool rating analysis (secondary outcome measure)

All participants agreed (> 3 on the Likert scale) on parameters 2–4 on the rating analysis. 10/11 participants agreed that the tool was easy to use and 9/11 enjoyed using the tool (Table 1).

## Discussion

This study adds to previous evidence supporting the use of CTA in orthopedic training (Bhattacharyya et al. 2017). The tool utilizes written and audiovisual information simultaneously to describe each step of antegrade femoral intramedullary nailing. The learner is equipped with knowledge on the technical steps, cognitive decision-making behind performing each technical step, and potential errors and solutions involved in this procedure.

This study showed substantially significantly better “post-test” scores in the group using the IFINCTA tool compared with the control group. Although there were improvements in both groups between the pre-test and post-test, the difference was much greater in the IFINCTA group compared with the control group. Our analysis rejected the null hypothesis and demonstrated that the IFINCTA is an effective tool to improve the cognitive understanding of an antegrade femoral intramedullary nailing procedure for novice learners. It is also superior compared with a standard operative technique manual, which is the gold-standard resource for learning surgical procedures prior to operating on patients.

Previous research has demonstrated benefits and a training effect of the “Touch Surgery™” application to teach femoral intramedullary nailing (Sugand et al. 2015a, 2015b). “Touch Surgery™” uses computerized animations and written information to teach procedural steps. There are no defined cognitive decision points and description of specific errors and solutions in this application. Furthermore, our tool has video clips (with superimposed audio voiceovers) recorded in an actual operating theatre demonstrating the entire procedure in real-time. The IFINCTA help the learners to truly appreciate the technical challenges in each procedural step involved and the cognitive reasoning behind performing each technical step, while also bringing to their attention potential errors and solutions using simultaneous written and audiovisual multimodal multimedia modalities. This makes our tool both educationally novel and technologically innovative.

Studies have highlighted the effectiveness of VR and cadaveric simulation to teach orthopedic trauma procedures (Leong et al. 2008, Sugand et al. 2015a). However (Karam et al. 2013), these are expensive and not readily accessible to trainees. Our CTA tool is web based and easily accessible. It allows repeated sustained practice, which is key in simulation training. Using this tool prior to attending the operating theatre will provide learners a sound understanding of the technical steps and the decision-making involved in this procedure. This will reduce their initial steep phase of the learning curve and allow more effective utilization of operating theatre training time. This is key in the current environment of reduced training hours (Chikwe 2004) available to achieve competency and can pave the way for CTA-based learning of complex surgical procedures in the future.

The subjective assessment of the tool indicated that the entire intervention group agreed that the tool aided their understanding of the procedure and was easy and enjoyable to use. The participants believed that this tool is a useful adjunct to learning in the operating room and they would prefer to use our tool prior to operating on patients.

### Strengths and limitations

The strengths of this study are: it is a prospective, double-blind randomized controlled trial with a comparable intervention and control group. We used a sound modified Delphi technique to design the IFINCTA tool. This tool has demonstrated significant benefits to improve the cognitive understanding of the procedure for novice students.

Our goal is for learners to use this tool prior to attending the operating theatre to give them a robust foundation in the understanding of this procedure, which will complement their learning in theatre. The limitation of this study is that we have not yet analyzed the impact of this tool to improve operating theatre performance (i.e., transfer validity), which carries its own ethical implications. We were obliged to demonstrate the ability of our CTA in a simulation setting to improve cognitive understanding prior to using it in the operating theatre, which will form part of our future work. Another potential limitation was that gender differences amongst the participants or the experts was not accounted for in this study.

### Application

With an ever-increasing rise of orthopedic trauma workload (Kehoe et al. 2015) and reduced training hours (Chikwe 2004), simulation-training techniques have increasingly been encouraged (Robbins et al. 2010). We have designed a user-friendly, accessible CTA learning tool that has demonstrated significant benefits in improving the knowledge of novice learners in antegrade femoral intramedullary nailing. We believe that similar CTA learning tools can be used in other orthopedic sub-specialties, thereby increasing its impact within orthopedic training.

## Conclusion

The IFINCTA tool has shown significant benefits in the cognitive understanding of novices in antegrade femoral intramedullary nailing. We recommend using our user-friendly, online tool to provide a strong foundation and a shorter cognitive learning curve for novices prior to starting their apprenticeship training in the operating theatre.

## Notes

Study and tool design: RB, KS, RB, and CMG. Data collection and analysis: RB, KS, and BA. CTA tool Delphi: IS, RB. and CMG. Writing and editing the manuscript: RB, KS, RB, and CMG.

Thanks are offered to Mr Victor Lesk for his help in designing the wizard tool.

*Acta* thanks Li Felländer-Tsai and Christian Krettek for help with peer review of this study.

## References

[CIT0001] Arora S, Aggarwal R, Sirimanna P, Moran A, Grantcharov T, Kneebone R, Sevdalis N, Darzi A Mental practice enhances surgical technical skills: a randomized controlled study. Ann Surg 2011; 253(2): 265–70. 21245669 10.1097/SLA.0b013e318207a789

[CIT0002] Bhattacharyya R, Davidson D J, Sugand K, Bartlett M J, Bhattacharya R, Gupte C M Knee arthroscopy simulation: a randomized controlled trial evaluatingthe effectiveness of the Imperial Knee Arthroscopy Cognitive Task Analysis (IKACTA) Tool. J Bone Joint Surg Am 2017; 99(19): e103. 28976437 10.2106/JBJS.17.00190

[CIT0003] BOTA Collaborators, Rashid M S. An audit of clinical training exposure amongst junior doctors working in trauma & orthopaedic surgery in 101 hospitals in the United Kingdom. BMC Med Educ 2018; 18(1): 1. 10.1186/s12909-017-1038-5 PMC5749005 29291730

[CIT0004] Camp C L, Krych A J, Stuart M J, Regnier T D, Mills K M, Turner N S Improving resident performance in knee arthroscopy: a prospective value assessment of simulators and cadaveric skills laboratories. J Bone Joint Surg Am 2016; 98(3): 220–5. 26842412 10.2106/JBJS.O.00440

[CIT0005] Cannon W D, Garrett W E, Hunter R E, Sweeney H J, Eckhoff D G, Nicandri G T, Hutchinson M R, Johnson D D, Bisson L J, Bedi A, Hill J A, Koh J L, Reinig K D Improving residency training in arthroscopic knee surgery with use of a virtual-reality simulator: a randomized blinded study. J Bone Joint Surg Am 2014; 96(21): 1798–806. 25378507 10.2106/JBJS.N.00058

[CIT0006] Chikwe J No time to train the surgeons. BMJ 2004; 328(7437): 418–419. 14976074 10.1136/bmj.328.7437.418 PMC344249

[CIT0007] Flin R, Youngson G, Yule S How do surgeons make intraoperative decisions? Qual Saf Health Care 2007; 16(3): 235–9. 17545353 10.1136/qshc.2006.020743 PMC2464983

[CIT0008] Karam M D, Pedowitz R A, Natividad H, Murray J, Marsh J L Current and future use of surgical skills training laboratories in orthopaedic resident education: a national survey. J Bone Joint Surg Am 2013; 95(1): e4. 23283381 10.2106/JBJS.L.00177

[CIT0009] Kehoe A, Smith J E, Edwards A, Yates D, Lecky F The changing face of major trauma in the UK. Emerg Med J 2015;32(12):911–15. 26598629 10.1136/emermed-2015-205265 PMC4717354

[CIT0010] Leong J J H, Leff D R, Das A, Aggarwal R, Reilly P, Atkinson H D E, Emery R J, Darzi A W Validation of orthopaedic bench models for trauma surgery. J Bone Joint Surg Br 2008; 90(7): 958–65. 18591610 10.1302/0301-620X.90B7.20230

[CIT0011] Luker K R, Sullivan M E, Peyre S E, Sherman R, Grunwald T The use of a cognitive task analysis-based multimedia program to teach surgical decision making in flexor tendon repair. Am J Surg 2008; 195(1): 11–15. 18082536 10.1016/j.amjsurg.2007.08.052

[CIT0012] Philibert I, Friedmann P, Williams W T, ACGME Work Group on Resident Duty Hours. Accreditation Council for Graduate Medical Education. New requirements for resident duty hours. JAMA 2002; 288(9): 1112–1114. 12204081 10.1001/jama.288.9.1112

[CIT0013] Rebolledo B J, Hammann-Scala J, Leali A, Ranawat A S Arthroscopy skills development with a surgical simulator: a comparative study in orthopaedic surgery residents. Am J Sports Med 2015; 43(6): 1526–9. 25769535 10.1177/0363546515574064

[CIT0014] RAD1382 Consensus Statement. The impact of EWTD on delivery of surgical services: a consensus statement. Association of Surgeons of Great Britain and Ireland; 2008 ewtd_consensus_statement_rad1382_.pdf.

[CIT0015] Robbins L, Bostrom M, Craig E, Sculco T P Proposals for change in orthopaedic education: recommendations from an orthopaedic residency directors’ peer forum. J Bone Joint Surg Am 2010; 92(1): 245–9. 20048120 10.2106/JBJS.I.00210

[CIT0016] Smink D S, Peyre S E, Soybel D I, Tavakkolizadeh A, Vernon A H, Anastakis D J Utilization of a cognitive task analysis for laparoscopic appendectomy to identify differentiated intraoperative teaching objectives. Am J Surg 2012; 203(4): 540–5. 22325336 10.1016/j.amjsurg.2011.11.002

[CIT0017] Spencer F Teaching and measuring surgical techniques: the technical evaluation of competence. Bull Am Coll Surg. 1978; 63(3): 9–12.

[CIT0018] Sugand K, Akhtar K, Khatri C, Cobb J, Gupte C Training effect of a virtual reality haptics-enabled dynamic hip screw simulator. Acta Orthop2015a; 86(6): 695–701. 26168925 10.3109/17453674.2015.1071111 PMC4750769

[CIT0019] Sugand K, Mawkin M, Gupte C Validating Touch SurgeryTM: A cognitive task simulation and rehearsal app for intramedullary femoral nailing. Injury2015b; 46(11): 2212–16. 26094504 10.1016/j.injury.2015.05.013

[CIT0020] Sullivan M E, Ortega A, Wasserberg N, Kaufman H, Nyquist J, Clark R Assessing the teaching of procedural skills: can cognitive task analysis add to our traditional teaching methods? Am J Surg 2008; 195(1): 20–3. 18082538 10.1016/j.amjsurg.2007.08.051

[CIT0021] Tomlinson J E, Yiasemidou M, Watts A L, Roberts D J H, Timothy J Cadaveric spinal surgery simulation: a comparison of cadaver types. Glob Spine J 2016; 6(4): 357–61. 10.1055/s-0035-1563724 PMC4868577 27190738

[CIT0022] Wallace L, Raison N, Ghumman F, Moran A, Dasgupta P, Ahmed K Cognitive training: how can it be adapted for surgical education? Surgeon. 2017; 15(4): 231–9. 27658665 10.1016/j.surge.2016.08.003

[CIT0023] Wingfield L R, Kulendran M, Chow A, Nehme J, Purkayastha S Cognitive task analysis: bringing olympic athlete style training tosurgical education. Surg Innov 2015; 22(4): 406–17. 25392150 10.1177/1553350614556364

